# Supplementary material for: Functional neurological symptoms occur commonly in healthy adults: implications for the pathophysiology of FND
Source: CNS Spectr. 2026 May 20;31(1):e13. doi: 10.1017/S1092852926100972 (PMC13732586; doi:10.1017/S1092852926100972)
Supplement: Supplementary file 1 [file S1092852926100972sup001.pdf]

Functional Neurological Experiences Questionnaire

David D. G. Palmer, Mark J. Edwards, Jason B. Mattingley

1. Have you ever had trouble moving a body part in the way you wanted to (including swallowing) which got worse the harder you tried, or seemed to work better when you were not concentrating on it?
   This includes both trouble getting a body part to move, and a body part moving in ways you didn't want or intend.

   ☐ Yes ☐ No

If Yes, please answer 1a, 1b, and 1c below, otherwise continue to question 2.

1a. How many times has this happened in your life?

- *Count each time a new symptom (or symptoms) began as a different instance.*
- *If a symptom was coming and going over short periods, count it as one instance.*
- *If a symptom resolved completely for a substantial amount of time then came back, count it as a different instance.*

Number of times	Select one
Only once	○
2 or 3 times	○
4 – 6 times	○
7 – 10 times	○
More than 10 times	○

1b. How long did the longest of these symptoms last for?
Count a symptom that was coming and going over short timeframes as one instance of the symptom.

Number of times	Select one
Less than a minute	◯
A few minutes	◯
More than a few minutes, but less than an hour	◯
More than an hour, but less than a day	◯
One day to one week	◯
One week to one month	◯
One month to three months	◯
Three months to a year	◯
More than a year	◯

1c. At their worst, how much did these symptoms impact on your work, education, or social life?

Number of times	Select one
Not at all	◯
Slightly *(eg, felt uncomfortable at work or school, or while socialising)*	◯
A little *(made work, education, or socialising difficult)*	◯
Quite a lot *(required up to a few days off work or school, or limited ability to socialise in certain situations)*	◯
A lot *(required more than a few days off work or school, or significantly limited ability to socialise)*	◯

2. Have you ever had “phantom” sensations that did not occur related to another medical condition?
This includes both numbness and feelings of things that aren't there, eg pins and needles, vibrations, or wetness.

☐ Yes ☐ No

If Yes, please answer 2a, 2b, and 2c below, otherwise continue to question 3.

2a. How many times has this happened in your life?
- *Count each time a new symptom (or symptoms) began as a different instance.*
- *If a symptom was coming and going over short periods, count it as one instance.*
- *If a symptom resolved completely for a substantial amount of time then came back, count it as a different instance.*

Number of times	Select one
Only once	◯
2 or 3 times	◯
4 – 6 times	◯
7 – 10 times	◯
More than 10 times	◯

2b. How long did the longest of these symptoms last for?
Count a symptom that was coming and going over short timeframes as one instance of the symptom.

Number of times	Select one
Less than a minute	◯
A few minutes	◯
More than a few minutes, but less than an hour	◯
More than an hour, but less than a day	◯
One day to one week	◯
One week to one month	◯
One month to three months	◯
Three months to a year	◯
More than a year	◯

2c. At their worst, how much did these symptoms impact on your work, education, or social life?

Number of times	Select one
Not at all	◯
Slightly *(eg, felt uncomfortable at work or school, or while socialising)*	◯
A little *(made work, education, or socialising difficult)*	◯
Quite a lot *(required up to a few days off work or school, or limited ability to socialise in certain situations)*	◯
A lot *(required more than a few days off work or school, or significantly limited ability to socialise)*	◯

3. Have you ever had dissociation (derealization or depersonalisation)?
This can include feeling like being in a dream while you are awake, like you are not quite in your own body, like you are not quite in the world, or that it is as though there is a veil between you and the world.

☐ Yes ☐ No

If Yes, please answer 3a, 3b, and 3c below, otherwise continue to question 3.

3a. How many times has this happened in your life?

- *Count each time a new symptom (or symptoms) began as a different instance.*
- *If a symptom was coming and going over short periods, count it as one instance.*
- *If a symptom resolved completely for a substantial amount of time then came back, count it as a different instance.*

Number of times	Select one
Only once	◯
2 or 3 times	◯
4 – 6 times	◯
7 – 10 times	◯
More than 10 times	◯

3b. How long did the longest of these symptoms last for?

Count a symptom that was coming and going over short timeframes as one instance of the symptom.

Number of times	Select one
Less than a minute	◯
A few minutes	◯
More than a few minutes, but less than an hour	◯
More than an hour, but less than a day	◯
One day to one week	◯
One week to one month	◯
One month to three months	◯
Three months to a year	◯
More than a year	◯

3c. At their worst, how much did these symptoms impact on your work, education, or social life?

Number of times	Select one
Not at all	◯
Slightly *(eg, felt uncomfortable at work or school, or while socialising)*	◯
A little *(made work, education, or socialising difficult)*	◯
Quite a lot *(required up to a few days off work or school, or limited ability to socialise in certain situations)*	◯
A lot *(required more than a few days off work or school, or significantly limited ability to socialise)*	◯
